# Identification of a Leaf Cuticular Wax Biosynthesis Gene *BrCER2* in Chinese Cabbage (*Brassica rapa* L. ssp. *pekinensis*)

**DOI:** 10.3390/plants14243831

**Published:** 2025-12-16

**Authors:** Yunshuai Huang, Xiaoyu Bai, Wenlong Ying, Yanbing Wang, Chaofeng Yang, Mujun Huang, Liai Xu, Huihui Fang, Jianguo Wu, Yunxiang Zang

**Affiliations:** Key Laboratory of Quality and Safety Control for Subtropical Fruit and Vegetable, Ministry of Agriculture and Rural Affairs, Collaborative Innovation Center for Efficient and Green Production of Agriculture in Mountainous Areas of Zhejiang Province, College of Horticulture Science, Zhejiang A&F University, Hangzhou 311300, China; 20230109@zafu.edu.cn (Y.H.); 13255718026@163.com (X.B.); 18268855100@163.com (W.Y.); wangyanbing95@163.com (Y.W.); f13767873445@163.com (C.Y.); hmj1220442201@163.com (M.H.); 20210035@zafu.edu.cn (L.X.); fanghh@zafu.edu.cn (H.F.); jianguowu@zafu.edu.cn (J.W.)

**Keywords:** Chinese cabbage, glossy trait, cuticular wax, BrKCS6, BrCER2

## Abstract

Glossy appearance is a critical trait that affects the appearance quality and marketability of leafy vegetables, including Chinese cabbage. The glossy trait is primarily associated with cuticular wax. Although several genes involved in cuticular wax biosynthesis have been characterized in Chinese cabbage, the regulatory relationships among them remain unclear. In this study, we identified a glossy mutant, *glossy leaf4* (*gl4*), and cuticular wax crystals in the *gl4* mutant were obviously reduced. Genetic analysis indicated that the glossy phenotype in the *gl4* mutant appears to be controlled by a single recessive gene. Using a bulked segregant analysis coupled with next-generation sequencing (BSA-seq) and map-based cloning methods, the *AtCER2* homologous gene *BrCER2* was identified as the candidate gene. *BrCER2* was expressed in various tissues, and BrCER2-GFP was localized in the endoplasmic reticulum (ER). Furthermore, BrCER2 could interact with BrKCS6 in the ER, and the expression levels of some wax biosynthesis-related genes were decreased in the *gl4* mutant. Our overall results provide insights about the role of BrCER2 in wax biosynthesis through ER localization and interaction with BrKCS6 in Chinese cabbage.

## 1. Introduction

Chinese cabbage (*Brassica rapa* L. ssp. *pekinensis*) is one of the cruciferous vegetable crops with the largest cultivation area and consumption in China. Plant cuticular wax is a hydrophobic barrier that appears as a glaucous frost-like substance on the surface of plant tissues, known as wax powder in cruciferous vegetable crops [1]. Cuticular wax not only enhances the plant’s resistance to stress, such as drought, high temperature, and diseases and pests, but also determines the appearance quality and commercial value [2,3,4]. For cabbage-type vegetable crops with leaves or flowering stalks as their product organs, those without wax powder have a fresh appearance and good commercial traits, which are more favored by consumers. Cuticular wax is a mixture composed of various substances, and its inheritance is controlled by multiple genes [3]. Research about cuticular wax formation has been extensively investigated in *Arabidopsis* and other model plants [5]. However, the corresponding research about cuticular wax remains relatively limited in Chinese cabbage.

The epidermal wax of plants is primarily composed of very-long-chain fatty acids (VLCFAs) and their derivatives, cyclic compounds, and certain secondary metabolites. Its biosynthetic pathway can be divided into three major steps: de novo fatty acid synthesis, VLCFA elongation, and wax derivative formation. In *Arabidopsis*, de novo fatty acid synthesis is catalyzed by the plastid fatty acid synthase complex (FAS), catalyzing the production of C16 and C18 fatty acids. CER8/LACS1 further converts them into C16 and C18 fatty acyl-CoA esters [6,7,8]. In maize, *ZmENR1*, encoding a plastid-localized enoyl-acyl carrier protein (ACP) reductase, is involved in the de novo fatty acid biosynthesis [9]. The VLCFAs (up to C28) are subsequently synthesized by the fatty acid elongase (FAE) complex, which comprises four core enzymatic components: β-ketoacyl-CoA synthase (KCS), β-ketoacyl-CoA reductase (KCR), β-hydroxyacyl-CoA dehydratase (HCD), and enoyl-CoA reductase (ECR) [10]. For chain elongation beyond C28, CER2 and its homologs are essential for coordinating the extension process [11,12]. Furthermore, the CER2-KCS6 module can collectively regulate pollen hydration and male sterility in rice and *Arabidopsis* [13,14]. Then, VLCFAs are subsequently channeled into two major pathways of the decarbonylation pathway and the acyl reduction pathway to generate additional wax components [3,15]. In *Arabidopsis*, CER1 is a key enzyme in the decarbonylation pathway and interacts with CER3 and CYTB5s. CER1 and CER3 serve as core components in the biosynthesis of very-long-chain alkanes, while CYTB5s is the electron donor that is necessary for the enzymatic activity of CER1 [16,17]. The acyl reduction pathway involves *WSD1*, responsible for wax ester biosynthesis, and *CER4*, essential for primary alcohol formation [18,19]. A recent study reported that *SOH1* encodes a putative aldehyde reductase and cooperates with *CER3* to establish a novel two-step reductive synthesis pathway for primary alcohol synthesis [20].

In cruciferous vegetable crops, several cuticular wax-related genes have been cloned using the glossy leaf materials; most of the cloned genes encode key enzymes in the cuticular wax biosynthesis pathway. In *Brassica oleracea*, the candidate genes of *Cgl1* and *Cgl2* are the homologs of *Arabidopsis CER1* and *CER4*, respectively, and the corresponding mutants exhibit glossy and brilliant green phenotypes [21,22,23]. In Chinese cabbage, *BrWAX2* and *BrWDM1*, encoding the *Arabidopsis* CER1 and CER4 homologous proteins, respectively, were also cloned by utilizing glossy mutants [24,25]. To date, CER2/CER2-LIKE genes and a few FAE complex components have been identified in cruciferous vegetable crops. In Chinese cabbage and broccoli, the candidate genes of *BrWAX1*/*BrCER2* and *BoGL5* were identified as the *Arabidopsis CER2* homologous gene; the coding sequence mutation of *BrCER2* and *BoGL5* was responsible for the glossy leaf and stem phenotypes [26,27,28]. In addition, *BrWAX3* and *BrKCS6* encode β-ketoacyl-CoA synthases (KCSs) of the FAE complex, which participate in VLCFA biosynthesis [29,30]. In *Arabidopsis*, the CER2/CER2-like proteins can interact with BrKCS6 to regulate pollen hydration, and AtKCS3 functions as a negative regulator in stem wax biosynthesis by interacting with AtKCS6 and reducing its activity [31]. However, knowledge about the molecular relationships between these wax biosynthesis regulators is still limited in Chinese cabbage.

In this study, we identified and characterized a glossy leaf mutant *gl4* in Chinese cabbage. Through BSA-seq and map-based cloning, we identified *BraA01g015290.3C* (*BrCER2*) as the candidate gene underlying the phenotype and analyzed its function through expression, localization, and interaction assays.

## 2. Results

### 2.1. The gl4 Mutant Shows a Glossy Phenotype

**Figure 1:**
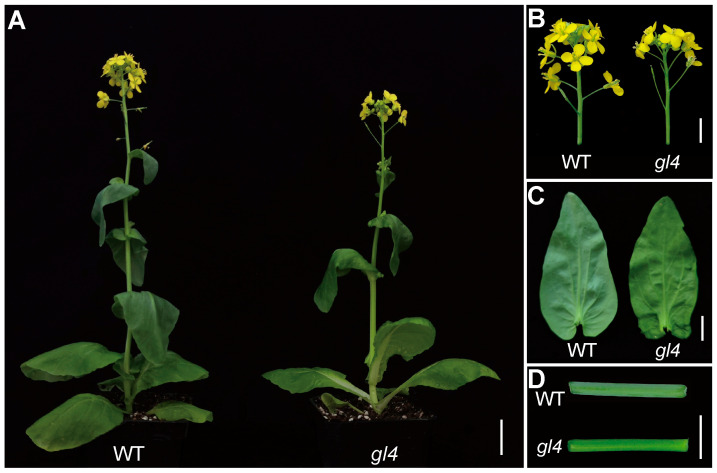
Phenotypic characterization of the wild-type and *gl4* mutant. (**A**) Morphologies of wild-type (WT) and *gl4* mutant plants at the bolting and blooming stage. Scale bar, 5 cm. (**B**) Inflorescence phenotypes of the WT and *gl4* mutant. Scale bar, 1 cm. (**C**) Leaf phenotypes of the WT and *gl4* mutant. Scale bar, 1 cm. (**D**) Stem phenotypes of the WT and *gl4* mutant. Scale bar, 1 cm.

To identify genetic factors regulating leaf glossiness in Chinese cabbage, we identified a glossy mutant named *glossy leaf 4* (*gl4*) from the inbred line Br74. Compared with waxy wild-type plants at the bolting and blooming stage, the *gl4* mutant showed a glossy phenotype in many aerial organs, such as the leaves, stems, and flower buds (Figure 1).

**Figure 2:**
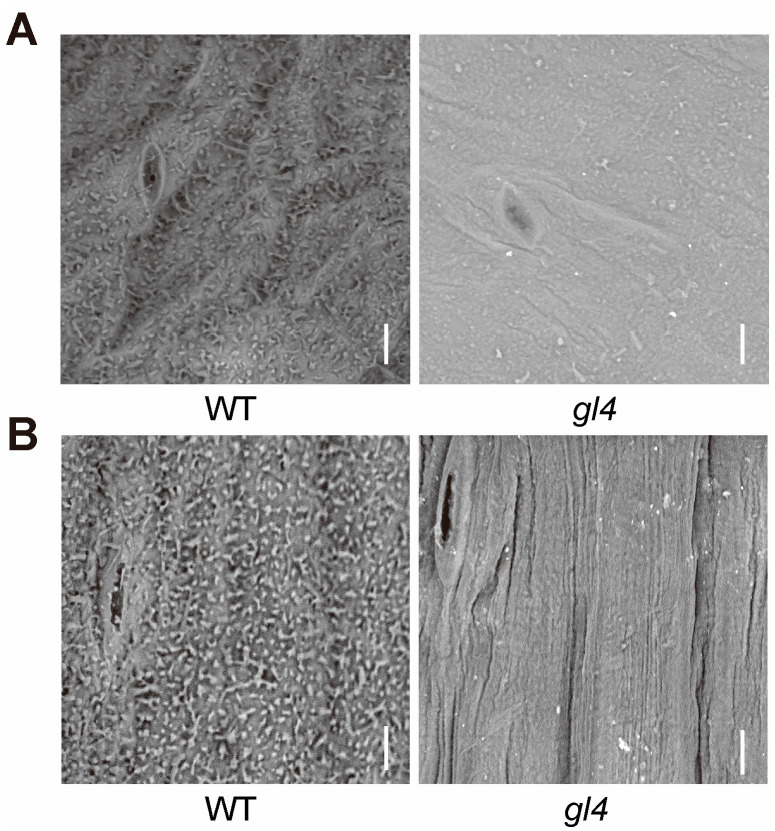
The microscopic structure of cuticular wax in the wild-type (WT) and *gl4* mutant. (**A**) High-resolution imaging using a scanning electron microscope of the leaves of the WT and *gl4* mutant. Scale bar, 5 μm. (**B**) High-resolution imaging using a scanning electron microscope of the stems of the WT and *gl4* mutant. Scale bar, 5 μm.

To investigate the cause of the glossy phenotype in the *gl4* mutant, we observed the microscopic structure of cuticular wax in leaves and stems by a scanning electron microscope (SEM). The SEM analysis showed that the wild-type had many wax crystals on the surface of the leaf and stem, and wax crystals were mainly columnar in the wild-type (Figure 2A,B). But, *gl4* had fewer wax crystals on the surface of the leaf and stem (Figure 2A,B). These results suggest that the reduction in cuticular wax is associated with the glossy phenotype of the *gl4* mutant.

### 2.2. The Cuticle Permeability of the gl4 Mutant Is Decreased

**Figure 3:**
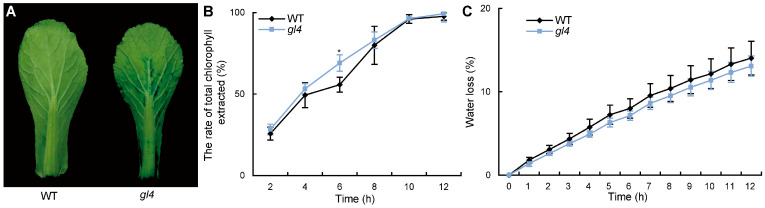
Cuticle permeability of the wild-type (WT) and *gl4* mutant. (**A**) Toluidine blue staining pattern of the WT and *gl4* mutant. (**B**) The rate of total chlorophyll extracted in the WT and *gl4* mutant. (**C**) Water loss rates of leaves in the WT and *gl4* mutant. Values in (**B**,**C**) are means ± SD (*n* ≥ 3). * *p* < 0.05 compared with the wild-type by Student’s *t*-tests.

The changes in cuticle composition are closely related to leaf permeability. To test the difference between wild-type and *gl4* in leaf permeability, we performed the toluidine blue (TB) staining assays. The leaf of the wild-type was barely stained, but the leaf of the *gl4* mutant was mildly stained (Figure 3A). Then, we also performed the chlorophyll extraction rate assay and water loss rate analysis. The results showed that the chlorophyll extraction rate of the *gl4* mutant was higher than that of the wild-type at 6 h, and the water loss rate of the wild-type was similar to that of the *gl4* mutant (Figure 3B,C, Table S1). These results indicated that the cuticle permeability of the *gl4* mutant is reduced.

### 2.3. Fine Mapping of the Candidate Gene

To isolate the causal gene in the *gl4* mutant, we first performed genetic analysis. We crossed the *gl4* mutant with the wild-type and generated an F_2_ population. The F_1_ plants were waxy and similar to the wild-type, and the ratio of the 162 waxy plants and 52 glossy plants was close to 3:1 (*χ*^2^ = 0.056 < *χ*^2^_[df = 1, *p* = 0.05]_ = 3.84, *p* > 0.05) in the F_2_ population. These results indicate that the *gl4* phenotype was controlled by a single recessive gene.

**Figure 4:**
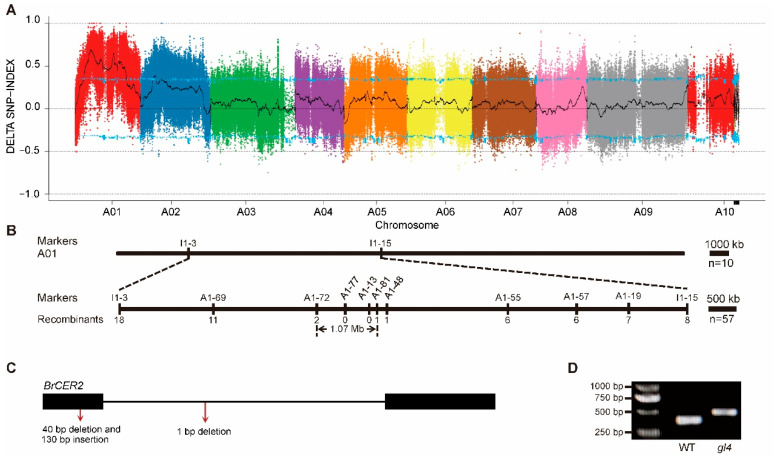
Map-based cloning of the candidate gene. (**A**) Distribution of SNP indexes on the 10 chromosomes in BSA-seq analysis. A total of 1000 permutation tests are performed, and the blue line indicates the threshold line at the 95% confidence level. (**B**) Fine mapping of the candidate gene. The markers and numbers of recombinants are indicated above and below the filled bars, respectively. (**C**) Gene structure of *BrCER2* (*BraA01g015290.3C*) and the mutation site of the *gl4* mutant. The black boxes and lines indicate exons and introns, respectively. The red arrows indicate the mutation sites in the *gl4* mutant. (**D**) Verification of the difference between the wild-type and *gl4* mutant in the genomic locus of *BrCER2* using a pair of markers.

To clone the mutant gene in the *gl4* mutant, we performed the BSA-seq assay. Firstly, we generated an F_2_ population from a cross between the *gl4* mutant and the non-heading Chinese cabbage inbred line Bc17 with a waxy leaf. In the F_2_ population, we selected 25 F_2_ plants with the *gl4* phenotype (glossy leaf) and 25 F_2_ plants with the Bc17 phenotype (waxy leaf) to construct a *gl4* phenotype mixed pool and a Bc17 phenotype mixed pool, respectively. Then, the *gl4* phenotype pool, the Bc17 phenotype pool, the *gl4* mutant, and Bc17 underwent whole-genome re-sequencing. The index of SNP/InDel and ΔSNP/InDel-index was calculated in Bc17, the *gl4* mutant, the *gl4* phenotype pool, and the Bc17 phenotype pool. From BSA-seq analysis, the ΔSNP-index of the 4.43 Mb–12.54 Mb region on chromosome A01 was significantly higher than the blue threshold line (Figure 4A). Therefore, the mutant gene was located in the 4.43 Mb–12.54 Mb region on chromosome A01. To further narrow the candidate region, we used 57 glossy plants for fine mapping the candidate gene. Finally, the mutant gene was limited to a 1.07 Mb region between markers A1-72 and A1-81 on chromosome A01 (Figure 4B). In the candidate region, *BraA01g015290.3C* was homologous to *AtCER2* in *Arabidopsis thaliana*. Compared to the wild-type, the first exon of *BraA01g015290.3C* in the *gl4* mutant contained a 40 bp deletion and a 130 bp insertion, leading to a frame shift and premature termination, and the first intron of *BraA01g015290.3C* in the *gl4* mutant had a 1 bp deletion, resulting in no change in coding sequence (Figure 4C and Figure S1). A 40 bp deletion and a 130 bp insertion were further confirmed by sequencing and PCR analysis (Figure 4D and Figure S1). Therefore, *BraA01g015290.3C* was selected as the candidate gene and named *BrCER2*.

### 2.4. Expression Pattern and Subcellular Localization of BrCER2

**Figure 5:**
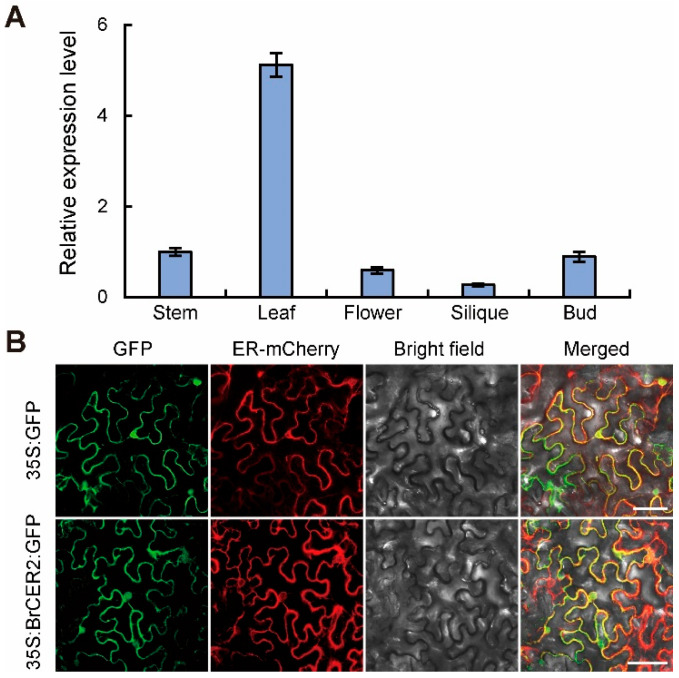
Expression pattern and subcellular localization of BrCER2. (**A**) The qRT-PCR analysis of the *BrCER2* expression level in different tissues of wild-type plants. The expression level of *BrCER2* in the stem was defined as “1”. (**B**) Subcellular localization of BrCER2-GFP fusion protein in *N. benthamiana* leaves. ER-rk CD3-959 fusing to mCherry protein was used as the ER localization marker. GFP protein was used as the control. Scale bar, 25 μm. Values in (**A**) are means ± SD (*n* = 3).

To investigate the biological function of *BrCER2*, we performed quantitative real-time PCR (qRT-PCR) analysis using various tissues. The qRT-PCR analysis result showed that *BrCER2* was expressed in many tissues and was highly expressed in leaves (Figure 5A). To determine the subcellular localization of the BrCER2 protein, we constructed a BrCER2-GFP fusion protein and transformed it into *N. benthamiana* leaves. Transient expression of the BrCER2-GFP fusion protein assay show that BrCER2-GFP is localized to the ER (Figure 5B).

### 2.5. Expression of Wax-Related Genes Is Down-Regulated in the gl4 Mutant

**Figure 6:**
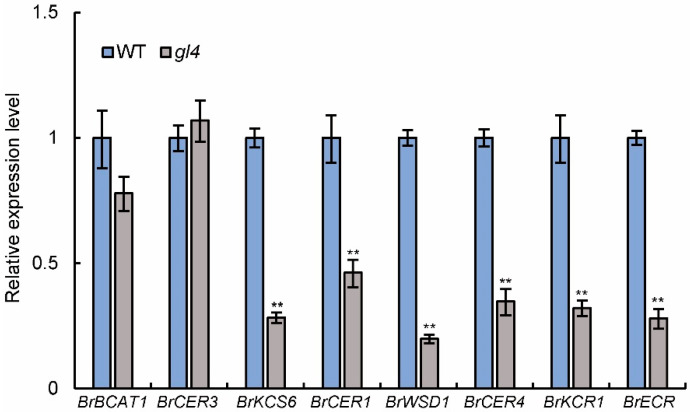
The expression levels of wax-related genes in the wild-type and *gl4* mutant. Quantitative RT-PCR analysis of the expression of *BrBCAT1*, *BrCER3*, *BrKCS6*, *BrCER1*, *BrWSD1*, *BrCER4*, *BrKCR1*, and *BrECR* in the leaves of the wild-type (WT) and *gl4* mutant. Values are means ± SD (*n* = 3). ** *p* < 0.01 compared with the wild-type by Student’s *t*-tests.

To analyze whether the *BrCER2* mutation affects transcription levels of wax-related genes, we examined the expression level of various wax-related genes in the leaves of the *gl4* mutant and wild-type plants at the bolting and blooming stages by qRT-PCR analysis. The expression level of wax biosynthesis-related genes differed between the *gl4* mutant and wild-type (Figure 6). Compared with the wild-type, the expression levels of VLCFA biosynthesis genes, including *BrKCS6*, *BrKCR1*, and *BrECR*, were significantly reduced in the *gl4* mutant (Figure 6). In addition, the transcript levels of the alkane-forming pathway and alcohol-forming pathway genes, including *BrCER1*, *BrWSD1*, and *BrCER4*, were reduced in the *gl4* mutant (Figure 6). These results suggest that the *BrCER2* mutation might influence the normal expression of wax-related genes.

### 2.6. BrCER2 Interacts with BrKCS6

**Figure 7:**
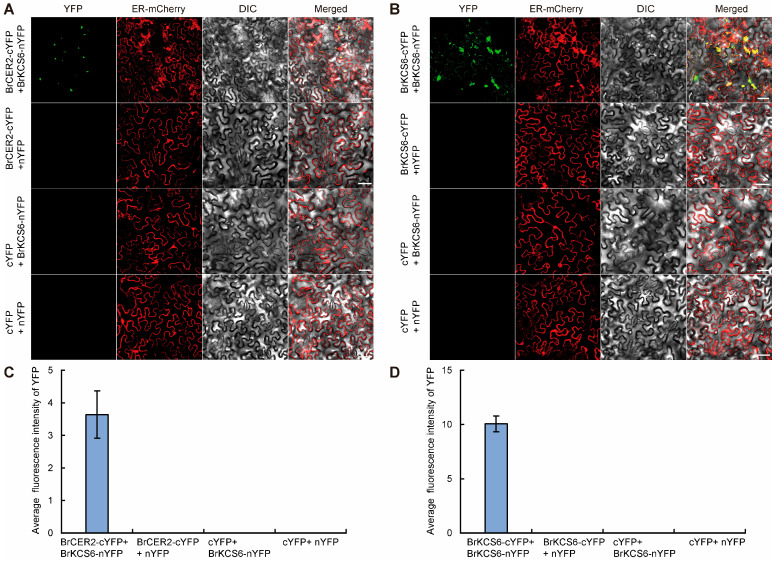
BrCER2 interacts with BrKCS6. (**A**) Bimolecular fluorescence complementation (BiFC) assays showing that BrCER2 interacts with BrKCS6. Scale bars, 50 μm. (**B**) BiFC assays showing that BrKCS6 interacts with BrKCS6. Scale bars, 50 μm. (**C**,**D**) Average fluorescence intensity of the YFP protein assay in (**A**,**B**) using ImageJ 1.53e software. Values are means ± SD (n = 3).

CER2 can interact with KCS6 in rice and *Arabidopsis*, and the *BrKCS6* mutation conferred a glossy phenotype in Chinese cabbage [13,14,29]. Therefore, we speculated that BrCER2 may interact with BrKCS6 to regulate the glossy phenotype in Chinese cabbage. Then, we performed a bimolecular fluorescence complementation (BiFC) assay to test the interaction between BrCER2 and BrKCS6 in *N. benthamiana* leaf cells. BrCER2 was fused with the C terminus of the yellow fluorescent protein (BrCER2-cYFP), and BrKCS6 was fused with the N terminus of the yellow fluorescent protein (BrKCS6-nYFP). A strong yellow fluorescent protein signal was observed in *N. benthamiana* leaf cells when BrCER2-cYFP was co-expressed with BrKCS6-nYFP, but no fluorescent protein signal was observed in other co-expressed combinations, including the BrCER2-cYFP/nYFP empty vector, BrKCS6-nYFP/cYFP empty vector, and cYFP/nYFP empty vectors (Figure 7A,C). And, the interaction position of BrCER2 and BrKCS6 was localized to the ER (Figure 7A). We also found that BrCER2 and BrKCS6 can interact with themselves, respectively, and BrKCS6 also functions in the ER (Figure 7B,D and Figure S2). These results show that BrCER2 can interact with BrKCS6 in Chinese cabbage.

## 3. Discussion

Leaves are the product organs in Chinese cabbage, and the glossy green phenotype of leaves is a crucial aspect of appearance and commercial quality. But, knowledge about gene cloning and the molecular mechanism of wax regulators is quite limited in Chinese cabbage. In this study, we identified a *gl4* mutant with a glossy leaf in Chinese cabbage. The reduction in cuticular wax crystals resulted in a glossy phenotype in the *gl4* mutant. The toluidine blue staining assay and chlorophyll leaching assay indicated that the leaf permeability increased less in the *gl4* mutant. Based on BSA-seq analysis and the map-based cloning assay, *BraA01g015290.3C* was found to be the homolog of *AtCER2* and identified as the candidate gene. Furthermore, BrCER2 was localized to the ER and interacted with BrKCS6. Our findings enhanced the understanding of *BrCER2* molecular function in regulating the glossy leaf trait in Chinese cabbage.

*AtCER2* belongs to the BAHD acyltransferase family and is involved in VLCFA elongation beyond C28 [12]. BrCER2 shared 74.1% identity with AtCER2, and BrCER2 may have a similar function as AtCER2 in VLCFA elongation. *BrCER2* is responsible for C28 fatty acid elongation [27]. Furthermore, BrCER2 was localized in the ER (Figure 5B), which is consistent with the action site of the FAE complex in VLCFA elongation. In Chinese cabbage, the *gl4* mutant, HN19-G material, and 08A235-2 material had a 40 bp deletion and a 130 bp insertion in the first exon of *BrCER2* [27,28]. The *BrCER2* of HN19-G material was inserted by a partial LINE-1 retrotransposon (*BrLINE1-RUP*) sequence [27]. The formation mechanism of the first exon sequence variation in the *BrCER2* of the *gl4* mutant, HN19-G material, and 08A235-2 material may be similar in Chinese cabbage. But, the *gl4* mutant is not the same material as HN19-G and 08A235-2 because the *gl4* mutant has a unique 1 bp deletion in the intron (Figure 4C). In addition, the function of *BrCER2* in the glossy phenotype regulation needs transgenic verification in Chinese cabbage. Some recent studies have reported that mutations in *BrMYB31*, *BrBCAT1*, and *BrKCS6* are responsible for the glossy phenotype and that these mutants exhibit a few surface wax crystals in the leaf along with significantly increased cuticle permeability in Chinese cabbage [2,29,32]. But, we found that the cuticle permeability in the *gl4* mutant increased less compared with that in the wild-type using the TB staining assay, chlorophyll extraction rate assay, and water loss rate assay (Figure 3). So, the *gl4* mutant is a good genetic resource for quality breeding in Chinese cabbage. Furthermore, the coding sequence mutation of *BrCER2* could be used for designing the molecular marker for marker-assisted selection (MAS) breeding in Chinese cabbage.

CER2 and CER2-LIKE proteins have been characterized in some plant species, including *Arabidopsis*, rice, broccoli, and Chinese cabbage [26,27,33,34,35]. However, CER2 proteins may have divergence of gene function and expression level in the various tissues of different plant species. The levels of C30 primary alcohol and C29 aldehydes were dramatically decreased in the *cer2* mutant in *Arabidopsis*, but the level of C30 primary alcohols showed no significant difference in the *BrCER2* mutant in Chinese cabbage, and the level of C29 aldehydes increased in the *BoCER2* mutant in broccoli [26,27,33]. So, the function of *CER2* may have diverged in *Arabidopsis*, Chinese cabbage, and broccoli. In *Arabidopsis*, *CER2* was only expressed in the epidermis of young siliques and stems, resulting in the *cer2* mutant with glossy stems, and their phenotype and wax components had no difference in leaves [33,36]. And, the *gl4* mutant, HN19-G material, and 08A235-2 materials had glossy traits in various tissues (Figure 1), including leaves, stems, and flowers [27,28]. Similarly, *BoGL5*/*BoCER2* was also highly expressed in leaves, and the corresponding mutant also showed a glossy appearance in various tissues [26]. Therefore, the different phenotypes between Chinese cabbage and *Arabidopsis* in leaves may be due to the different expression pattern of *BrCER2* in the leaves (Figure 5A). In addition, the total amounts of wax in the leaf were far lower than those in the stem in *Arabidopsis*, and the different wax amounts between leaf and stem may contribute to phenotypic differences [31]. For example, the *AtKCS6* mutant and *AtKCS3* overexpression lines obviously reduced total wax amounts in the leaf and stem, but only the stems exhibited a glossy phenotype [31]. SEM analysis showed that the *gl4* mutant had few wax crystals in leaves and stems compared to the wild-type (Figure 2A,B). Therefore, it is needed to further investigate the regulatory mechanism of *CER2* at the transcriptional level among *Arabidopsis* and Chinese cabbage in future studies.

CER2 and its homologs and FAE complex play important roles in VLCFA elongation, and CER2/CER2-LIKE proteins could interact with different FAE complex components [37]. CER2 could interact with the FAE complex component KCS6 in *Arabidopsis* and rice [13,14,34]. CER2/CER2-LIKE proteins could influence KCS6 activity in the VLCFA elongation pathway [11,38]. The CER2-KCS6 module plays important roles in different growth and development processes. The CER2-KCS6 module could affect pollen hydration in *Arabidopsis*, and it also influenced leaf cuticular wax synthesis and male sterility in rice [13,14,34]. Recently, *AtCER19* encodes acetyl-CoA carboxylase1 and catalyzes the synthesis of malonyl-CoA. And, AtCER19 interacts with AtCER2 and affects AtCER2 activity in VLCFA elongation. AtCER2 may act as an adaptor to mediate the formation of the AtCER19-AtCER2-AtKCS6 complex [39]. However, the relationship between CER2 and KCS6 is unclear in *Brassica* vegetable crops, including Chinese cabbage. In Chinese cabbage, *BrKCS6*, encoding 3-ketoacyl-CoA synthases, was identified as the candidate gene of *wdm9* and *wdm10*, and the two mutants had a glossy appearance in various tissues, including leaves, stems, and siliques [29]. The glossy phenotype of the *BrKCS6* mutant was similar to that of the *gl4* mutant. The BiFC assay showed that a strong fluorescent signal was observed when BrCER2-cYFP was co-expressed with BrKCS6-nYFP in *N. benthamiana* leaf cells (Figure 7A). Thus, this interaction was conserved in Chinese cabbage. And, the expression levels of wax-related genes were changed in leaves of the *gl4* mutant (Figure 6). *BrKCS6*, *BrKCR1*, and *BrECR*, involving VLCFA biosynthesis genes, were significantly reduced; *BrCER1*, *BrWSD1*, and *BrCER4*, involving alkane-forming and alcohol-forming pathways, were also reduced in the *gl4* mutant (Figure 6). *BrKCS6* is the FAE complex component, and its mutant also affects the transcriptional level of wax-related genes [29]. Therefore, the role of *BrCER2* in VLCFA and wax biosynthesis further expanded our knowledge about CER2 proteins.

In conclusion, we identified a glossy leaf mutant *gl4* and revealed that *BraA01g015290.3C* (*BrCER2*) was identified as the candidate gene. BrCER2 encoded BAHD acyltransferase and interacted with BrKCS6 in the ER. The present findings provide a new insight for breeding bright leaf varieties and a better understanding of the wax biosynthesis pathway in Chinese cabbage. In addition, CER2 and CER2-LIKE proteins had an important role in the development of plant pollen. *AtCER2* and *AtCER2L2* were likely involved in pollen hydration, and the VLCFAs contents of the *cer2 cer2L2* double mutant were decreased in the pollen coat, resulting in defective hydration and male sterility [14]. In rice, OsCER26L was identified as an HMS1-interacting protein and played an important role in humidity-sensitive genic male sterility [13]. And, *BrCER2* was also expressed in the flower (Figure 5A). Thus, it is necessary to clarify the function of *BrCER2* in anther and pollen development in Chinese cabbage in future studies.

## 4. Materials and Methods

### 4.1. Plant Materials and Growth Conditions

The wild-type Br74 was a Chinese cabbage inbred line from the Kangaibaicai variety, and the *gl4* mutant was identified from Br74. The *gl4* mutant had a glossy phenotype, and wild-type plants had a waxy appearance in the bolting and blooming stages of the leaf, stem, and pistil. For genetic analysis, Br74 and *gl4* mutant were used to generate F_1_ plants and an F_2_ population. For BSA-seq analysis and gene mapping, the non-heading Chinese cabbage lines Bc17 and the *gl4* mutant were used as parents to generate the F_2_ population. The germinated seeds were transferred to a 4 °C refrigerator for 20 days of vernalization. The vernalized seeds were cultivated in a growth chamber maintained at 70% relative humidity, a photosynthetic photon flux density (PPFD) of 600 μmol m^−2^ s^−1^, a 16 h light/8 h dark photoperiod, and a constant temperature of 24 °C. After 25 days, plant materials were cultured in the plastic house at Zhejiang A&F University.

### 4.2. Scanning Electron Microscopy Analysis

The fresh leaves and stems of the wild-type and the *gl4* mutant were collected at the bolting stage. The leaves and stems were cut into 0.5 cm squares and dried in a vacuum freeze-drier for 36 h. The dehydrated samples were plated with gold using the ion sputter coater (SBC-12, KYKY, Beijing, China) for 40 s. And, the morphology of cuticular wax crystals was observed by the scanning electron microscope (TM4000 plus, Hitachi, Tokyo, Japan) at 15 kV.

### 4.3. Toluidine Blue (TB) Test

The toluidine blue test was performed using leaves at the bolting stage as described previously [40]. The fresh leaves of wild-type and *gl4* mutant plants were collected at the bolting stage. The fresh leaves were incubated in 0.05% (*w*/*v*) toluidine blue for 20 min and then washed with water to remove the toluidine blue from the leaf surface.

### 4.4. Chlorophyll Leaching Assay and Measurement of Water Loss

Chlorophyll leaching assay was performed using the leaves of the wild-type and *gl4* mutant at the bolting stage. Approximately 2 g of leaves was immersed in 30 mL of 80% ethanol at room temperature. The 3 mL aliquot of each sample was taken every hour. And, the chlorophyll amount in the aliquot was determined by measuring absorbance at 647 nm and 664 nm wavelength using a UV/VIS spectrophotometer (UV-5500, Shanghai METASH instruments company, Shanghai, China). The concentration and rate of total chlorophyll extracted were calculated as described previously [40]. The concentration of total chlorophyll was calculated using the following equation: total micromoles chlorophyll = 7.93 (A664)  +  19.53 (A647). The rate of total chlorophyll extracted was calculated by the percentage of the different times of chlorophyll over the total chlorophyll extracted after 24 h.

The leaves at the bolting stage were used to conduct water loss rate analysis. The leaves were dried at room temperature, weighed every one hour using a microbalance, and then dried at 65 °C until the dry weight remained constant. Total water content was calculated by the fresh weight minus the dry weight. The rate of water loss was calculated as the percentage of water loss over the total water.

### 4.5. BSA-Seq Analysis

For BSA-seq analysis, the F_2_ population was generated by a cross between the *gl4* mutant and the non-heading Chinese cabbage inbred line Bc17 with a waxy leaf. The waxy phenotypes of Bc17, *gl4* mutant, F_1_ plants, and F_2_ population were observed. Equal amounts of DNA from 25 F_2_ plants with the glossy leaf phenotype and 25 F_2_ plants with the waxy leaf phenotype were pooled to construct a glossy mixed pool and a waxy mixed pool, respectively. DNA from the two pools, Bc17 and the *gl4* mutant, was sequenced on an Illumina Hiseq^TM^ PE150 platform. These samples used the *B. rapa* genome v3.0 as the reference genome. The sequencing depth and coverage of four samples in the BSA-seq assay are listed in Table S2. A BWA (Burrows–Wheeler Aligner) was used to align the clean reads of each sample against the reference genome. The SNP index and INDEL index were calculated at each position of the glossy mixed pool and the waxy mixed pool according to the read depth information for homozygous SNPs/InDels. The ΔSNP/InDel-index of each SNP/InDel position was calculated by subtracting the SNP/InDel-index of the glossy mixed pool from the SNP/InDel-index of the waxy mixed pool. SNP/InDel and ΔSNP/InDel indices were analyzed following a previously described method [41]. The BSA-seq analysis was performed by the Novogene company (Peking, China).

### 4.6. Map-Based Cloning

For genetic analysis, an F_2_ population was generated from a cross between the *gl4* mutant and the wild-type Br74, and the ratio of the waxy and glossy phenotypes was obtained. For map-based cloning, we first generated an F_2_ population from a cross between the *gl4* mutant and the non-heading Chinese cabbage inbred line Bc17 with a waxy leaf, and ten F_2_ plants with the recessive glossy leaf phenotype were used for preliminary mapping. Then, 57 glossy leaf plants from the F_2_ population were selected for fine mapping. InDel/SSR markers were designed using Primer Premier 5.0 software according to the genomes of Chiifu (*B. rapa* cv. Chiifu genome v3.0, Chinese cabbage) and pak choi (*B. rapa* cv. Pak choi genome v1.0, non-heading Chinese cabbage) posted on the BRAD website. The polymorphism screening of InDel primers in the *gl4* mutant, Bc17, and the waxy and glossy pools was performed using polyacrylamide gel electrophoresis. The screened polymorphic InDel/SSR markers were used to fine-map the mutant gene. Molecular markers for fine mapping are listed in Table S3.

### 4.7. DNA, RNA Extraction, and qRT-PCR Analysis

Fresh leaves were harvested for DNA isolation using the cetyltrimethylammonium bromide (CTAB) method. For the expression pattern assay, the total RNA from fresh leaves, stems, flowers, siliques, and buds was extracted using the RNA prep Pure Plant Kit (TIANGEN, Peking, China). For the wax-related gene expression assay, the fresh leaves from the *gl4* mutant and Br74 were harvested and isolated from total RNA using the RNA prep Pure Plant Kit. A total of 1 μg of total RNA was used to synthesize first-strand complementary DNA (cDNA) using the Takara Prime Script 1st Strand cDNA Synthesis kit. The *BrACTIN* gene was used as an internal control, and the primers of wax biosynthesis-related genes were used as described previously [32]. Quantitative RT-PCR assays were performed on a qTOWER3/G real-time PCR machine (Jena, Germany) using a SYBR Premix Ex Taq^TM^ kit (Takara, Peking, China). The 2^-ΔΔCt^ method was used to calculate relative expression levels. The primers for the qRT-PCR assay are listed in Table S3.

### 4.8. Subcellular Localization of BrCER2 Protein

For the subcellular localization of BrCER2, the C-terminus of BrCER2 was fused to GFP protein in the *pCAMBIA1305.1* vector. The ER-rk CD3-959 fusing to mCherry protein was used as the ER localization marker. These constructs were transformed into the Agrobacterium strain EHA105. Then, EHA105 containing constructs were transformed into tobacco mesophyll cells, and the tobacco was incubated for 2 days at 28 °C. Fluorescence signals of GFP and mCherry protein were observed on a confocal laser scanning microscope (Olympus FV3000, Tokyo, Japan).

### 4.9. Bimolecular Fluorescence Complementation Assays

For BiFC assays, the full-length coding sequence of *BrCER2* and *BrKCS6* was inserted into *p2YN* and *p2YC* vectors, respectively. BiFC assays were conducted according to a previously described method [42]. These plasmids were transformed into the Agrobacterium strain EHA105, and different combinations of these constructs were co-transfected into tobacco mesophyll cells. And, the tobacco was incubated for 2 days at 28 °C. The ER-rk CD3-959 fusing to mCherry protein was used as the ER localization marker. Fluorescence signals of YFP and mCherry proteins were detected using the confocal laser scanning microscope (Olympus FV3000, Tokyo, Japan). The fluorescence intensity of the YFP protein assay was measured using ImageJ 1.53e software. Primers used for BiFC assays are listed in Table S3.

### 4.10. Statistical Analysis

Statistical analysis was calculated using IBM SPSS software 19.0. Significant differences were analyzed through Student’s *t*-test (* *p* < 0.05; ** *p* < 0.01).

## 5. Conclusions

In this study, we identified *BrCER2* and provided new insights about the function of *BrCER2* in glossy leaf regulation in Chinese cabbage. The glossy appearance of the *gl4* mutant is caused by the reduction in cuticular wax. Using BSA-seq analysis combined with a map-based cloning approach, *BrCER2* was identified as the candidate gene. *BrCER2* was expressed in various tissues, especially in leaves. The *BrCER2* mutation resulted in reducing the expression levels of some wax-related genes, including *BrKCS6*, *BrCER1*, and *BrWSD1*. Furthermore, BrCER2 was localized in the ER and could interact with BrKCS6 and itself. Our findings advance understandings of the function of *BrCER2* in the wax biosynthesis network in Chinese cabbage.

## Data Availability

The data used to support the findings of this study are available from the corresponding author upon request.

## Supplementary Materials

The following supporting information can be downloaded at https://www.mdpi.com/article/10.3390/plants14243831/s1, Figure S1. Sequence alignment of the BrCER2 in the wild-type and *gl4* mutant. Figure S2. BrCER2 interacts with BrCER2. Table S1. Water loss rates of leaves in the wild-type and *gl4* mutant. Table S2. The sequencing depth and coverage of four samples in the BSA-seq assay. Table S3. Primers used in this study.
