# Supplementary material for: Identification of a Leaf Cuticular Wax Biosynthesis Gene BrCER2 in Chinese Cabbage (Brassica rapa L. ssp. pekinensis)
Source: Plants (Basel). 2025 Dec 16;14(24):3831. doi: 10.3390/plants14243831 (PMC12736802; doi:10.3390/plants14243831)
Supplement: Supplementary file 1 [file plants-14-03831-s001.zip › plants-3961538-supplementary.pdf]

Supplementary Materials

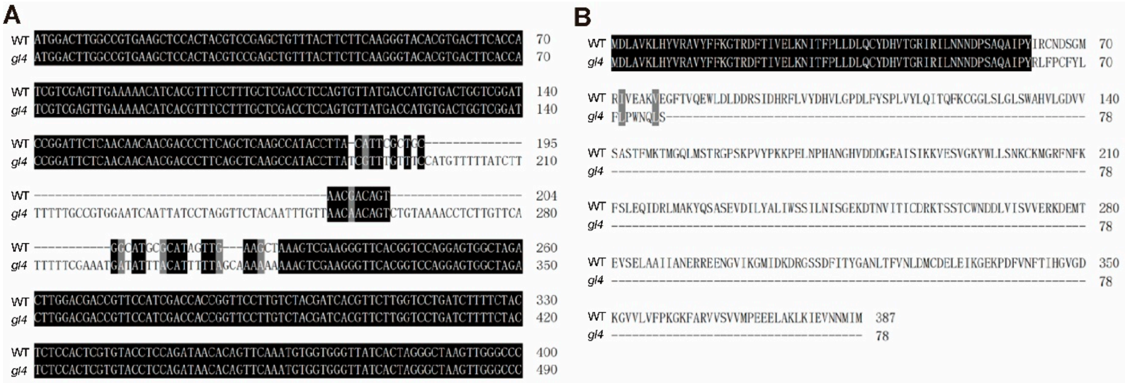

Figure S1. Sequence alignment of the BrCER2 in the wild-type and *gl4* mutant. (A) DNA sequence of the *BrCER2* locus in the in the wild type and *gl4* mutant. (B)Sequence alignment of the BrCER2 protein in the wild type and *gl4* mutant.

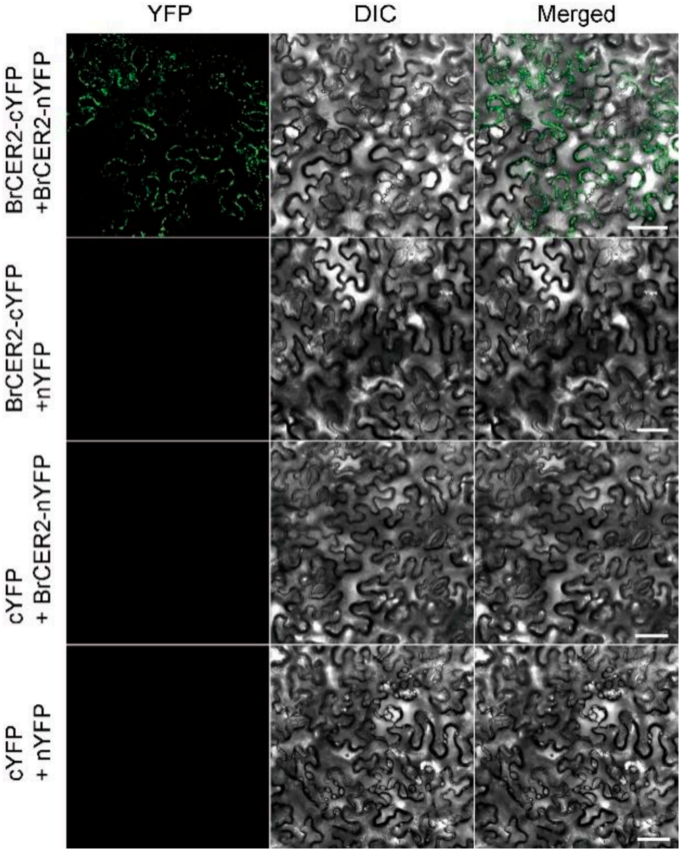

Figure S2. BrCER2 interacts with BrCER2. Bimolecular fluorescence complementation (BiFC) assays showing that BrCER2 interacts with BrCER2. Scale bars, 50 μm.

Table S1. Water-loss rates of leaves in the wild-type and *gl4* mutant

Time (h)	Water-loss rates of leaves in Wild-type (%)	Water-loss rates of leaves in *gl4*(%)	*p*-values
1	1.792 ± 0.330	1.381 ± 0.367	0.222
2	3.071 ± 0.495	2.576 ± 0.220	0.189
3	4.347 ± 0.650	3.771 ± 0.295	0.235
4	5.756 ± 0.934	4.908 ± 0.373	0.218
5	7.249 ± 1.150	6.300 ± 0.527	0.264
6	8.002 ± 1.172	7.142 ± 0.561	0.316
7	9.557 ± 1.402	8.615 ± 0.721	0.36
8	10.402 ± 1.562	9.526 ± 0.833	0.44
9	11.439 ± 1.675	10.532 ± 1.021	0.468
10	12.168 ± 1.793	11.371 ± 1.097	0.547
11	13.311 ± 1.946	12.326 ±1.199	0.497
12	14.037 ± 2.015	13.084 ± 1,206	0.521

Table S2. The sequencing depth and coverage of four samples in the BSA-seq assay

Sample	Mapped reads	Total reads	Mapping rate (%)	Average depth (X)	Coverage at least 1X (%)	Coverage at least 4X (%)
gl4	54229056	56092880	96.68	21.18	94.42	90.72
**Bc17**	58055801	60033608	96.71	23.58	89.69	84.93
**glossy mixed pool**	105905015	109197626	96.98	39.77	97.45	94.88
**waxy mixed pool**	103952450	107193108	96,98	39.22	96.71	93.54

Table S3. Primers used in this study

Purpose	Primer name	Primer sequence
**qRT-PCR**	qBrCER2-F	AACAACAACGACCCTTCAGC
	qBrCER2-R	GGAGGTACACGAGTGGAGAG
	qBrACTIN-F	CGAAACAACTTACAACTCC
	qBrACTIN-R	CTCTTTGCTCATACGGTCA
	qBrBCAT1-F	ATTGGGACAACCTCGGATTCA
	qBrBCAT1-R	TCCAGCAGCAGGGTTGAGC
	qBrCER3-F	CCATTGCTCTTTACCTCTGTCG

	qBrCER3-R	AGTTTTGAGCAGCGTTGTATTTG
	qBrKCS6-F	ATGGAACACTCTCGTCTCAACCT
	qBrKCS6-R	GATAACCAACTCTGCTTCGCTTC
	qBrCER1-F	TCCCTCGTATTACCCTTCTTGC
	qBrCER1-R	CATCAACACTCCGTCTGTCCTC
	qBrWSD1-F	GTCTAAACCCCTTTGGGAACTCC
	qBrWSD1-R	AAAGTCCAGCAATCAACCACCA
	qBrCER4-F	AGACGAGATTTTGGGGAAGGAC
	qBrCER4-R	CGTCCCCATTGACAACAGTAAC
	qBrKCR1-F	GCACTTACTTCAAATCCCAGCC
	qBrKCR1-R	CGGAGGAAGTAGATGTAGAGGGA
	qBrECR-F	GACTCTTCCTGTCTCTCCTGGG
	qBrECR-R	GAAGAGCGTGCGGTATGAGACT
**DNA mutation verification**	BrCER2-mutation-F	CGGATTCTCAACAACAACGA
	BrCER2-mutation-R	ATAGCAACTTTCTTCGTAAT
**Subcellular localization**	1305-BrCER2-GFP-F	CGGAGCTAGCTCTAGAGAAATGAAGGCAAGCCCAGT
	1305-BrCER2-GFP-R	TGCTCACCATGGATCCCGACACGAATGCTTCCAAAT
**BiFC assay**	P2YN-BrKCS6-F	CATTTACGAACGATAGTTAATTAAATGTCGCCTCCTAAAATGCC
	P2YN-BrKCS6-R	CACTGCCACCTCCTCCACTAGTTAGTTTGACAACTTCAGGGA
	P2YC-BrKCS6-F	CATTTACGAACGATAGTTAATTAAATGTCGCCTCCTAAAATGCC
	P2YC-BrKCS6-R	CACTGCCACCTCCTCCACTAGTTAGTTTGACAACTTCAGGGA
	P2YC-BrCER2-F	CATTTACGAACGATAGTTAATTAAATGGACTTGGCCGTGAAGCT
	P2YC-BrCER2-R	CACTGCCACCTCCTCCACTAGTCATAATCATATTATTCACCT
	P2YN-BrCER2-F	CATTTACGAACGATAGTTAATTAAATGGACTTGGCCGTGAAGCT
	P2YN-BrCER2-R	CACTGCCACCTCCTCCACTAGTCATAATCATATTATTCACCT
**Map-based cloning assay**	I1-3-F	TCTCTTCGTCATTATCATCT
	I1-3-R	ATCCAATGGTGTTGAAAGTA
	A1-69-F	CCTTTTGTCTGAAGATGATT
	A1-69-R	GAAGGACAGGTTCCATTGAT
	A1-72-F	TCCTCTGCTCAAATGAAATG
	A1-72-R	TCCAACATTTGTATCATCTG
	A1-77-F	TAAATGGACTGTAACAATGG
	A1-77-R	TGCCATAGTTTCCATAGATT
	A1-13-F	CTTTGGATTTGACCGTGCTA
	A1-13-R	GATGAAATCCTGTTTGTAAT
	A1-81-F	TTCCAGGTTATGATACTCCA
	A1-81-R	CGATAAAGCACCCAAGGCGG
	A1-48-F	AGGCAGGGGCAGATGAGGAG
	A1-48-R	ACTCAACATCCTTCCGCAAC

	A1-55-F	ATAAGGCAAGGAGGAAAACT
	A1-55-R	CGAGCGTTCTGATTTCATTA
	A1-57-F	CTTGCTTGCTTATGCCGTCC
	A1-57-R	TAAACGCACCAAAAAGACAA
	A1-19-F	TTTATTAGAGACTACACTTC
	A1-19-R	CATTGCGGAGTTTCTCATTC
	I1-15-F	GGCTGCTGATGAACAGAAGT
	I1-15-R	GCGTGCTGTTGGAAATAAAG

Accession Codes: Sequence data from this article can be found in the BRAD databases under the following accession numbers: *BrCER2* (BraA01g015290.3C); *BrKCS6* (BraA09g037930.3C); *BrBCAT1* (BraA06g006950.3C); *BrKCR1* (BraA02g017810.3C); *BrECR* (BraA09g046170.3C); *BrCER1* (BraA09g066480.3C); *BrCER3* (BraA02g012040.3C); *BrCER4* (BraA01g004350.3C); *BrWSD1* (BraA05g014990.3C).
